# Synthesis and Optoelectronic Characterization of Some Star-Shaped Oligomers with Benzene and Triphenylamine Cores

**DOI:** 10.5402/2012/976178

**Published:** 2012-08-22

**Authors:** Teofilia Ivan, Loredana Vacareanu, Mircea Grigoras

**Affiliations:** Electroactive Polymers Department, Institute of Macromolecular Chemistry “Petru Poni”, 41A Gr. Ghica Voda Alley, 700487 Iasi, Romania

## Abstract

Six star-shaped oligomers containing triphenylamine (**D1**–**D3**) and benzene unit (**D4**–**D6**) as cores have been synthesized by Wittig condensation or Heck coupling reaction using aromatic aldehydes and triphenylphosphonium salts or aromatic halogenated compounds with vinyl triphenylamine. All oligomers have well-defined molecular structure and high purity. Characterization of the oligomers was made by FT-IR, ^1^H-NMR spectroscopy, UV-Vis, and fluorescence spectroscopy. The electrochemical behavior was studied by cyclic voltammetry (CV). The cyclic voltammograms have revealed that oligomers undergo quasireversible or irreversible redox processes. The irreversible process is associated with electrochemical polymerization of oligomers by dimerization of unsubstituted triphenylamine groups. Thermal characterization was accomplished by TGA and DSC methods and evidenced that all oligomers were stable materials until 250°C and have formed stable molecular glasses after first heating scan.

## 1. Introduction

Conjugated oligomers with linear, highly branched and dendrimer structures form an important class of electro- and photoactive materials, investigated both in academic and industrial laboratories [1–9]. These architectures have advantages to offer molecules with a well-defined form and structure, a high chemical purity, and degree of order, being characterized by a polydispersity degree of one. It is important to note that the purity of materials is vital for the long-term stability of optoelectronic devices. Dendrimers can be obtained using laborious step-by-step synthesis in a convergent or divergent methodology [10]. The advantage of using small conjugated compounds is based on the possibility of tuning their photophysical properties by changing the chemical structure, for example, by introduction of side substituents, end-capping groups, insertion of certain specific functional groups, and by changing the oligomer length. Moreover, conjugated oligomers are used as model compounds for conducting polymers since their monodispersity, defectless structure, and better supramolecular organization in the solid state facilitate their experimental and theoretical investigations. The real interests for conjugated oligomers emerge also from interesting application such as active components in organic electronic or electrochemical devices, such as organic light emitting diodes (OLEDs) [11–15], photovoltaic cells [16, 17], optical power limiting [18], and field-effect transistors [19]. One of the important challenging goals of the conjugated oligomer chemistry is to develop new *π*-conjugated structures to investigate their photophysical and chemical properties, as well as to understand the structure-property relationship within such structures.

Triphenylamine (TPA) oligomers have been widely investigated for almost two decades because these compounds have shown excellent solubility, thermal and electrochemical stability, electron donating ability, and optoelectronic properties [9, 20]. They have been used most widely as the hole-transporting layer in EL devices, due to their amorphous film-forming ability and their high carrier mobility. They also can act as efficient dyes for sensitized solar cells [21]. Heck and Wittig reactions were extensively utilized to build the vinylene bond in each phenylenevinylene conjugation unit. The triphenylamine derivatives connected by the C–C double bond can be synthesized by Wittig and Heck reactions without air-sensitive catalyst or high temperatures and prolonged reaction time, and the structure similar to the p-phenylene moiety has the interesting photochemical and photophysical properties. In this paper, we report the synthesis, characterization, and photophysical properties of six representative conjugated star oligomers having trisubstituted 1,3,5-benzene and 4,4′,4′′-triphenylamine as the cores. The first five oligomers can be considered conjugated dendrimers of first generation while the sixth oligomer is a dendrimer of second generation. They have high fluorescence quantum yield, which indicats that these oligomers are candidates for the application in OLED as light emitting or photovoltaic materials. Relationship between chemical structure and optoelectronic properties of these compounds was investigated by spectral methods, such as UV-Vis and fluorescence spectroscopy, Cyclic Voltammetry, and thermal methods.

## 2. Experimental 

### 2.1. Materials and Instruments

1,3,5-tris (bromomethyl) benzene (97%, mp = 94–99°C), triphenylamine (98%), sodium t-butoxide (t-C_4_H_9_ONa), triethylamine, palladium (II) acetate, and tri (o-tolyl) phosphine were purchased from Aldrich and used as received. Dioxane and tetrahydrofuran (THF) were dried on sodium wire and distilled under nitrogen just before use. 4-Formyl triphenylamine [22] and 4,4′,4′′-triformyl triphenylamine [23–25] were obtained by formylation of TPA according to the reported methods. 4-Formyl-N,N′-bis(4-bromophenyl) aniline [26], tris (4-iodophenyl) amine [27] and vinyl triphenylamine [28] were synthesized and purified according to the literature procedures. Triphenylphosphonium salts were also obtained in a series of chemical reactions in our laboratory, using the starting compounds: 1,3,5-tris (bromomethyl) benzene, N,N-dimethylaniline, and anthracene. 1,3,5-Tris (methylene-triphenyl phosphonium bromide) benzene was prepared as described in literature [29]. 4-(N,N-Dimethylamino)benzyl(triphenyl)phosphonium iodine was synthesized according to the reported procedure [30]. 9-Anthrymethyl triphenylphosphonium chloride was synthesized starting from anthracene by three steps: formylation [31], followed by reduction to 9-anthrylmethanol [32], exchange the –OH with chlorine using SOCl_2_ as reagent [31], and finally, reaction with PPh_3_ [29].

The FT-IR spectra were recorded in KBr pellets on a DIGILAB-FTS 2000 spectrometer. UV-Vis and fluorescence measurements were carried out in solution using spectrophotometric grade solvents, on a Specord 200 spectrophotometer and Perkin Elmer LS 55 apparatus, respectively. ^1^H-NMR spectra were recorded at room temperature on a Bruker Avance DRX-400 spectrometer (400 MHz) as solutions in CDCl_3_, and chemical shifts are reported in ppm and referenced to TMS as internal standard. The cyclic voltammograms (CV) were recorded using a Bioanalytical System, Potentiostat-Galvanostat (BAS 100B/W) system. The electrochemical cell was equipped with three electrodes: a working electrode (Pt, Φ = 1.6 mm) or ITO-coated glass (platinum plate) with 0.15 × 0.5 cm^2^, an auxiliary electrode (platinum wire), and a reference electrode (consisted of a silver wire coated with AgCl). Before experiments, ITO-coated glass electrode was sonicated in a mixture of detergent and methanol for 5 min and then rinsed with a large amount of doubly distilled water. The reference electrode (Ag/AgCl) was calibrated at the beginning of the experiments by running the CV of ferrocene as the internal standard in an identical cell without any compound in the system (*E*_1/2_ = 0.425 V versus Ag/AgCl). Prior to each experiment, electrolyte solutions were deoxygenated by passing dry argon gas for 10 min. All measurements were performed at room temperature (25°C) under argon atmosphere. Melting points were determined with Electrothermal MEL-TEMP^R^ apparatus. Thermal gravimetric analyses (TGA) were performed by means of a STA 449 F1 Jupiter device, in N2 atmosphere, with a flow of 40 mL/min, and a heating speed of 10 K/min (30–900°C range). DSC measurements were performed with a Mettler DSC-12E apparatus, in nitrogen.

### 2.2. Synthesis of Arylenevinylene Oligomers by Wittig Method

**Figure 1:**
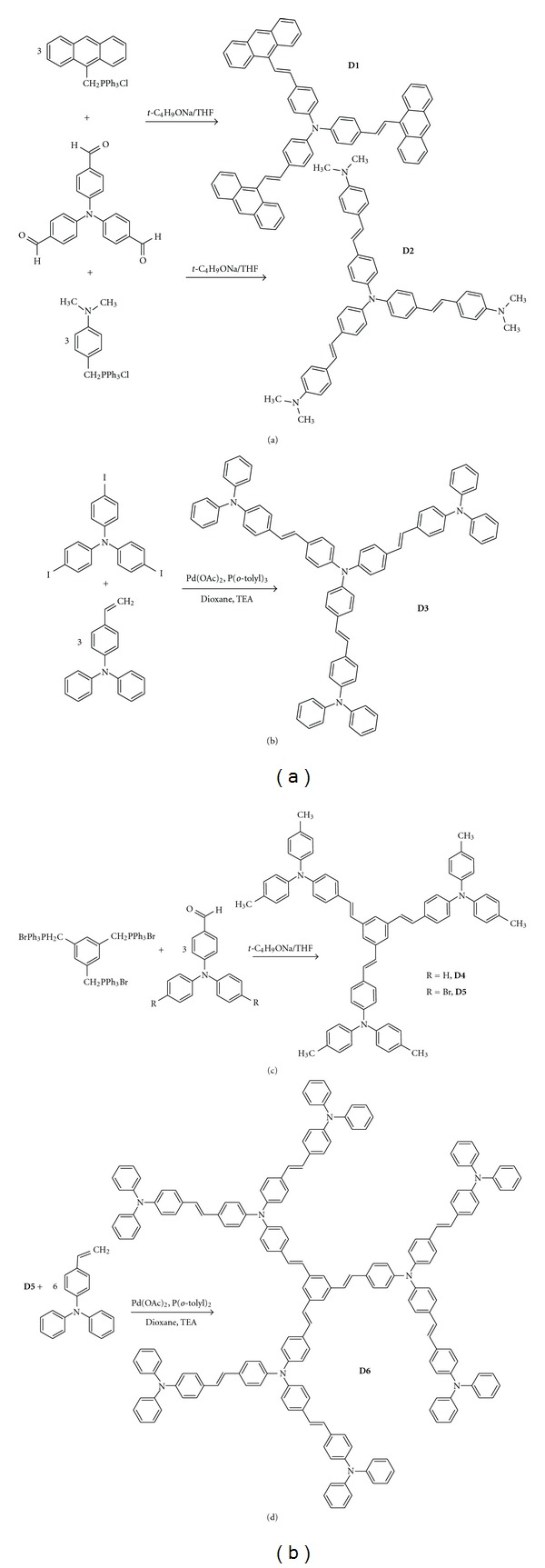
Synthesis of star-shaped oligomers D1–D6.

 Compounds **D1**, **D2**, **D4**, and **D5**, containing triphenylamine or benzene as cores, were synthesized by Wittig condensation starting from triphenylamine aldehydes with corresponding triphenylphosphonium salts (Figure 1). Aldehydes and phosphonium derivatives were dissolved in dry THF (10 mL) at 0°C and a base was added, and the reaction mixture was warmed to room temperature and stirred under N_2_ overnight. The reaction mixture was poured into water, filtered, and dried. The products were purified by column chromatography using dichloromethane/hexane (1/10). They were obtained as yellow powders with strong green-yellow fluorescence in diluted solution.

**D1**: to a solution of tris (4-formylphenyl) amine (0.15 g, 0.45 mmol) and 9-anthrymethyl triphenylphosphonium chloride (0.69 g, 1.4 mmol) in 10 mL THF, t-BuONa (0.17 g, 1.82 mmol) in 20 mL of dry THF was dropwise added to the resulting solution maintained at 0°C, then the reaction mixture was warmed to room temperature and stirred under N_2_ overnight. The reaction mixture was poured into water, filtered, and dried. The crude product was purified by flash column chromatography using dichloromethane/hexane (1 : 10) to give 0.21 g compound **D1 **(54.3%) as a yellow solid. Mp: 271-272°C.

FT-IR (cm^−1^, KBr): 3044–2923 (=C–H), 1597 (C=C), 1440, 1322 (C–N stretching vibration), 1176, 1016, 971 (out-of-plane bending vibration of *trans* –CH=CH–), 731, 508.

^1^H-NMR (CDCl_3_): 8.42–8.40 (t, 9H), 8.04–8.01 (m, 6H), 7.91–7.87 (d, 3H vinyl), 7.66–7.64 (d, 6H), 7.50–7.25 (m, 12 H), 7.32–7.25 (d, 6H), 6.98–6.94 (d, 3H vinyl).

Elemental analysis, %: Calculated for C_66_H_45_N (852.09 amu): C, 93.04; H, 5.32; N, 1.64. Found: C, 92.79; H, 5.42; N, 1.50.

**D2**: Tris (4-formylphenyl) amine (0.2 g, 0.60 mmol) and 4-(N,N-dimethylamino) benzyl (triphenyl) phosphonium iodine (0.98 g, 1.8 mmol) were dissolved in 10 mL of dry THF and t-BuONa (0.3 g, 3.12 mmol) in 20 mL of dry THF was added under cooling. The color solution changes, initially was mauve and finally changed to darkred. The reaction mixture was warmed to room temperature and stirred overnight under N_2_. The reaction mixture was poured into water, filtered, and dried. The crude product was purified by flash column chromatography using dichloromethane and hexane (1 : 10 v/v ratio). Yield = 0.245 g (59.5%) of **D2** as a yellow solid. Mp: 140–144°C. 

FT-IR (cm^−1^, KBr): 3440–3397 (N–H), 3020–2797 (=C–H), 1606 (C=C, conjugated phenyl groups), 1353, 1321 (C–N stretching vibration), 962 (out-of-plane bending vibration of *trans* –CH=CH–), 826, 543.

^1^H-NMR (CDCl_3_): 7.41–7.35 (m, 12H), 7.08–7.06 (d, 6H), 6.98–6.86 (m, 6H), 6.73 (d, 6H, vinyl), 2.99 (s, 18H, –CH_3_).

Elemental analysis, %: Calculated for C_48_H_48_N_4_ (680.935 amu) C, 84.67; H, 7.10; N, 8.23. Found: C, 83.25; H, 7.02; N, 8.11.

**D4**: 1,3,5-Tris (methylene-triphenyl phosphonium bromide) benzene (0.75 g, 0.65 mmol) and 4-formyl triphenylamine (0.53 g, 1.95 mmol) were dissolved in 10 mL of dry THF; t-BuONa (0.18 g, 1.95 mmol) in 20 mL of dry THF was added dropwise slowly to the resulting solution at 0°C, then the reaction mixture was warmed to room temperature and stirred overnight under N_2_. The reaction mixture was poured into water, filtered, and dried. The crude product was purified by flash column chromatography using dichloromethane/hexane as eluent, yield: 0.23 g (41.1%) of compound **D4** as yellow solid. Mp: 191°C. 

FT-IR (cm^−1^, KBr): 3438, 3034–2852 (=C–H), 1587 (C=C, conjugated phenyl groups), 1506, 1490, 1278, 1075, 1028, 960 (out-of-plane bending vibration of *trans* –CH=CH–), 753, 696.

^1^H-NMR (CDCl_3_): 7.49 (s, 3H), 7.42–7.38 (6H), 7.28–7.21 (m, 12H), 7.13–7.11 (m, 12H), 7.06–7.01 (m, 18H).

Elemental analysis, %: Calculated for C_66_H_51_N_3_ (886.15 amu) C, 89.46; H, 5.80; N, 4.74. Found: C, 88.18; H, 5.46; N, 4.38.

**D5**: A solution of 4-formyl-N,N′-bis (4-bromophenyl) aniline (0.56 g, 1.31 mmol) and 1,3,5-tris (methylene-triphenyl) phosphoniumbromide) benzene (0.5, 0.43 mmol) was dissolved in 10 mL of dry THF. The resulting solution was dropwise slowly added to 0.12 g (1.31 mmol) of t-BuONa in 20 mL of dry THF at 0°C, then the reaction mixture was warmed to room temperature and stirred overnight under N_2_. The mixture reaction was poured into water, filtered, and dried. The crude product was obtained as a yellow solid, yield: 0.26 g (44.1%). Mp: 142-143°C.

FT-IR (cm^−1^, KBr): 3447, 3028–2852 (=C–H), 1581 (C=C, conjugated phenyl groups), 1507, 1485, 1312, 1285, 1272, 1176, 1007, 961 (out-of-plane bending vibration of *trans* –CH=CH–), 819, 710, 508.

^1^H-NMR (CDCl_3_): 7.51 (s, 3H), 7.37–7.35 (18 H), 6.98–6.96 (18H), 6.91–6.87 (6H).

### 2.3. Synthesis of Arylenevinylene Oligomers by Heck Reaction

Oligomers **D3** and **D6** were synthesized by Heck coupling reaction of halogenated aromatic compounds with 4-vinyl triphenylamine. The mixture of compounds and catalyst palladium (II) acetate and tri (o-tolyl) phosphine in anhydrous dioxane/triethylamine mixture was stirred 24 h at 80–90°C under argon. The reaction mixture was poured into methanol, filtered, and dried. The resulting compounds were obtained as yellow powders.

**D3**: A round-bottomed flask (25 mL) oven dried and cooled under N_2_ atmosphere was introduced: tris (4-iodophenyl) amine (0.5 g, 0.80 mmol), 4-vinyltriphenylamine (0.65 g, 2.4 mmol), Pd(OAc)_2_ (0.04 mmol), and tri (o-tolyl phosphine) (0.2 mmol) in 10 mL dioxane and 5 mL triethylamine. The reaction mixture was heated to 80–90°C overnight. The solution was poured into methanol the precipitate was filtered, washed with methanol, water and dried. The crude product is obtained as yellow solid (0.476 g). Yield = 56.6% of **D3**. Mp=257-258°C.

FT-IR (cm^−1^, KBr): 3026–2851 (=C–H), 1581, 1591 (C=C, conjugated phenyl groups), 1508, 1314, 1280, 959 (out-of-plane bending vibration of *trans* –CH=CH–), 752, 695 (C–H phenyl rings).

^1^H-NMR (CDCl_3_): 7.62–7.60 (3H, vinyl), 7.49–7.41 (t, 6H), 7.32–7.29 (m, 12H), 7.07–7.05 (d, 6H), 7.03–7.01 (m, 24H), 6.97–6.93 (d, 6H), 6.83–6.81 (3H, vinyl).

^13^C-NMR, CDCl_3_ 100 MHz: 147.77, 147.49, 147.11, 147.03, 146.35, 146.00, 138.25, 138.12, 132.75, 132.34, 131.59, 129.19, 127.19, 126.40, 125.83, 124.26, 123.78, 122.56, 77.33–77.69 (from CDCl_3_).

**D6**: Compound **D5 **(0.05 g, 0.04 mmol), 4-vinyltriphenylamine (0.08 g, 0.28 mmol), palladium (II) acetate (0.002 mmol), and tri (o-tolyl phosphine) (0.01 mmol) were stirred in dioxane (10 mL) and triethylamine (5 mL) and heated and stirred overnight at 80–90°C under argon. The reaction mixture was poured in methanol, filtered, washed, and dried. The resulting compound was obtained as a yellow solid with yield = 58.1% (0.036 g). Mp: 240°C.

FT-IR (cm^−1^, KBr): 3435, 3026–2852 (=C–H), 1583 (C=C, conjugated phenyl groups), 1507, 1484, 1312, 1283, 1071, 1007, 961 (out-of-plane bending vibration of *trans*–CH=CH–), 696, 508.

^1^H-NMR (CDCl_3_): 7.51 (s, 3H), 7.44–7.42 (d, 6H), 7.39–7.35 (m, 24H Ph and vinyl), 7.31–7.22 (m, 24H), 7.20–7.03 (m, 48H), 6.98-6.92 (m, 30H Ph and vinyl), 6.92–6.87 (6H).

## 3. Results and Discussion

Six star-shaped oligomers, **D1**–**D6**, with arylenevinylene structure have been synthesized by condensation of triphenylamine aldehydes with triphenylphosphonium salts and by Heck coupling reaction between aromatic halogenated compounds and 4-vinyl triphenylamine. The synthetic pathways and chemical structures of compounds are given in Figure 1.It had to be mentioned that syntheses of **D2** [33] and **D3** [34, 35], using another synthetic routes, were reported but only nonlinear optical properties were studied.

The FT-IR and ^1^H-NMR spectra in CDCl_3_ have confirmed the expected oligomer structures (see Experimental). The FT-IR spectra have shown the characteristic bands of the chromophore groups: 2923–3020 cm^−1^ attributed to aromatic (*ν*C–H); 1581–1606 cm^−1^ assigned to the phenyl rings (*ν*C=C). All oligomers have transarylenevinylene structure which is also confirmed by characteristic IR absorption peak at 959–971 cm^−1^ corresponding to the out-of-plane bending vibration of HC=CH.

### 3.1. Photophysical Properties

**Figure 2:**
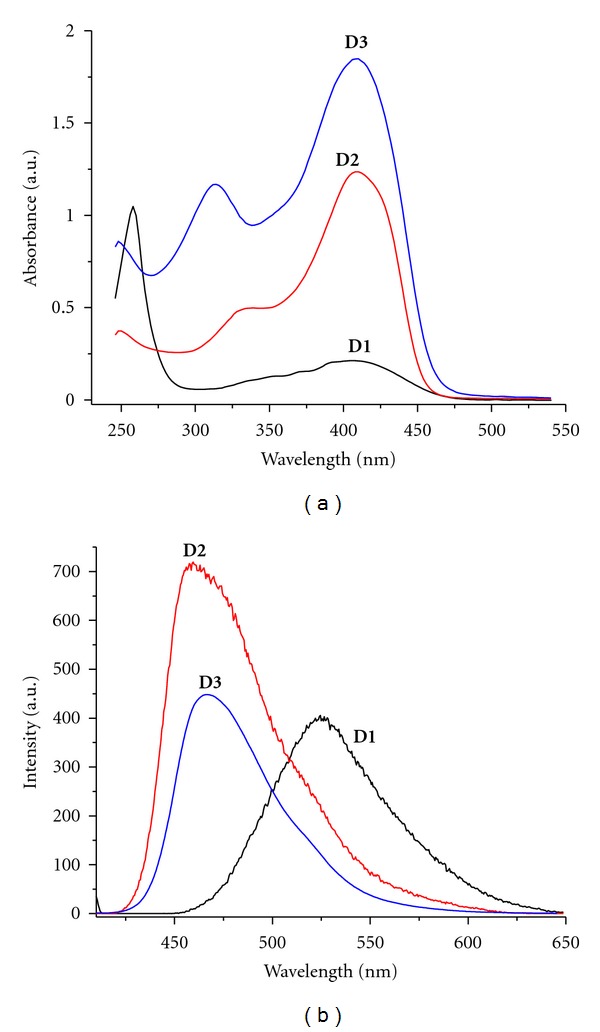
Absorption (a) and fluorescence (b) spectra of conjugated oligomers with triphenylamine core: **D1**, **D2**, **D3** in dilute chloroform solution (10^−5 ^M). Emission spectra were obtained upon excitation at the absorption maximum.

**Figure 3:**
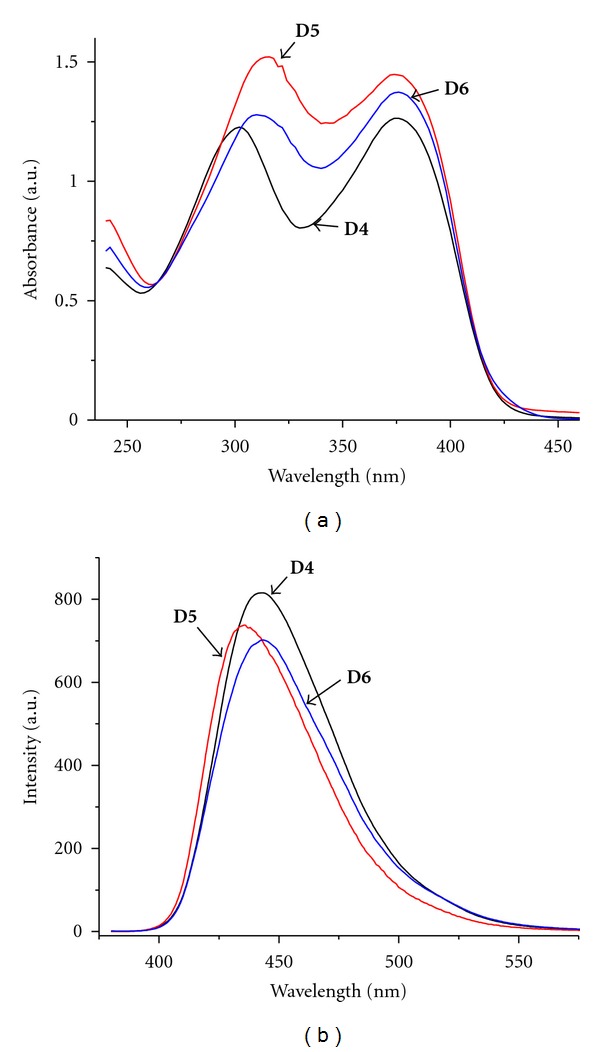
Absorption (a) and fluorescence (b) spectra of conjugated oligomers with benzene core: **D4**, **D5**, **D6**, in dilute chloroform solution (10^−5 ^M). Emission spectra were obtained upon excitation at the absorption maximum.

The UV-Vis absorption and photoluminescence spectra of oligomers are shown in Figures 2 and 3. All absorption spectra consist of two distinct absorption regions with maxima at 250–325 nm and 370–406 nm without any vibronic features. The first maximum is assigned to *π*-π* transition in aromatic rings while the second maximum is due to *π*-π* in all conjugated molecule.  Figure 2 presents the absorption and photoluminescence spectra of the **D1**, **D2**, and **D3 **oligomers, recorded in chloroform. 

All three oligomers have a triphenylamine ramification node, three vinylene conjugated spacers and three anthracene, triphenylamine or p-N,N-dimethyl benzene as peripheral groups. The absorption spectrum shape of **D1** is characteristic for substituted vinyl anthracene (three groups) [36], while the absorption of triphenylamine core is covered. The absorption spectrum of 9-vinylanthracene (in CHCl_3_) has two absorption regions with vibronic structure (260 nm and 355, 372, and 390 nm) being bathochromically shifted compared with anthracene. The bathochrome shift of absorption spectrum of **D1** in comparison with vinylanthracenes is assigned to contribution of TPA core that by nitrogen central atom does not interrupt the conjugation between the three branches. The absorption spectrum of **D3** shows a maximum absorption at 406 nm. 4,4-Bis (diphenylamino) stilbene, model compound of a branch, exhibits a maximum absorption peak at 370 nm (in CHCl_3_ solution) [37]. The strong bathochrome shift of *λ*_max⁡_ of **D3** (36 nm) in comparison with 4,4-bis (diphenylamino) stilbene model could be explained by participation of nitrogen central atom to conjugation, the conjugation between the three branches being uninterrupted. The absorption curves of **D2** and **D3** are very similar showing two absorption maxima at ~325 nm and 404 nm for **D2**, and 308, 330 nm and 406 nm for **D3**. The absorption maximum of **D3** is bathochromatically shifted, related to **D2**, phenyl groups having a stronger donor effect in comparison with methyl ones.

Diluted solutions of oligomers in CHCl_3_ are characterized by a yellowish-green fluorescence. The emission spectra show a bathochromic shift in the order: **D1** > **D3** > **D2** (Table 1) and this may be explained by increasing of conjugation length in oligomers. A dependence of fluorescence of **D1**, **D2**, and** D3 **upon excitation wavelengths was not observed. The absorption data of all oligomers are summarized in Table 1.

Figure 3 shows absorption and photoluminescence spectra of the oligomers having 1,3,5-trisubstituted benzene ring as core (**D4**, **D5**, **D6**), in chloroform solution. All oligomers display two absorption peaks in the range of 302–316 nm and 374–376 nm, respectively. The first absorption peak situated in the low wavelength region, 302–316 nm, was attributed to the triphenylamine moiety. The second absorption peak at 374–376 nm was ascribed to the TPA-vinylene-modified benzene unit. As compared with **D1**–**D3** oligomers, the *λ*_max⁡_ of **D4**–**D6** is blue-shifted; the metha substituted benzene core interrupts the conjugation between the three branches.

A red-shifting of the absorption maxima was observed in the series **D4** < **D5 **< **D6 **due to the increasing in the same order of the effective conjugation length of the branches. For **D5**, bromine atoms have an electron withdrawing effect and *λ*_max⁡_ is lower than unsubstituted **D4**. Compounds **D4** and **D6** are dendrimers of first, and respectively, second generation. With increasing of the generation of dendrimers, the effective conjugation increases [38]. 

Figure 3(b) illustrated the PL spectra of oligomers in dilute chloroform solutions. For the emission spectra in dilute chloroform solutions, **D4**, **D5**, and **D6 **display similar behaviors. The red-shift of the emission peak of **D6 **from that of **D4 **and** D5 **show that there is obvious *π*-π* delocalization and a significant increase of the effective conjugation length with the increase of the generation of dendrimers. A comparison of UV and PL spectra of **D3** and **D4** oligomers shows that triphenylamine is a better conjugation node than benzene core.

### 3.2. Electrochemical Properties

**Figure 4:**
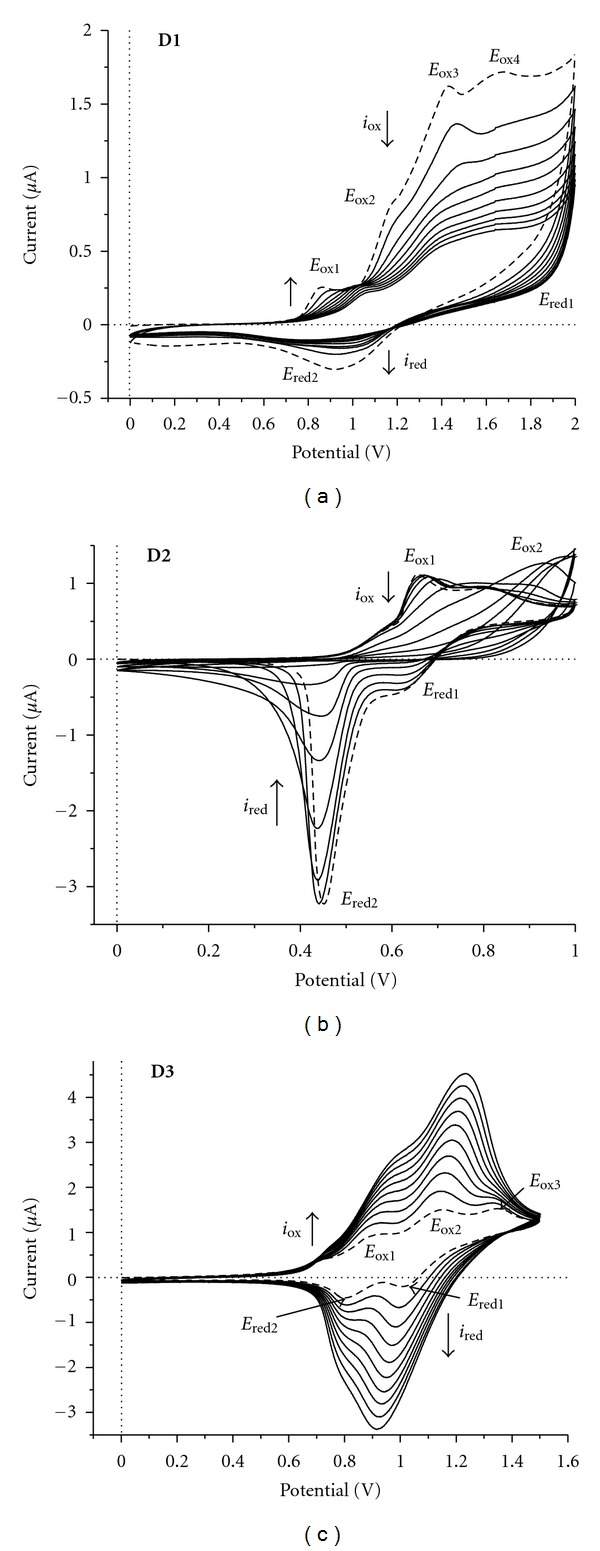
Cyclic voltammograms for compound **D1** (a), **D2 **(b), **D3 **(c), 10^−3 ^M, using Bu_4_NBF_4_ as support electrolyte (10^-1 ^M). Scan rate: 50 mV/s between 0.0 and 2.01 V versus Ag/AgCl.

The electrochemical characteristics of the star-shaped molecules were investigated by cyclic voltammetry. Generally, increasing the chain length and number of electroactive sites involves a higher electronic delocalization which may cause differences in redox behavior of the derivatives. Cyclic voltammograms of compounds have been determined in CH_2_Cl_2_ with tetrabutylammonium tetrafluoroborate as electrolyte. All measurements were carried out at room temperature and argon atmosphere. Representative cyclic voltammograms are shown in Figure 4. 

In general, all molecules undergo a quasireversible redox process arising from triphenylamine central unit followed by a series of quasireversible or irreversible oxidations originating from bridged aromatic peripheral moieties. 

Figure 4(a) shows the CV of **D1**. The first scan (dotted line) revealed four oxidation peaks and one reduction peak. First oxidation peak, occurring at *E*_ox1_ = 0.86 V, is caused by the formation of the TPA cation radical. The further oxidation process revealed three anodic peaks at *E*_ox2_ = 1.15 V, *E*_ox3_ = 1.43 V, and *E*_ox4_ = 1.67 V. These peaks can be attributed to the oxidation of the n-*π* conjugated system between triphenylamine and anthracene units at the ends. On the reverse scanning, one reduction peak can be observed located at *E*_red1_ = 0.94 V, which is very broad. The intensity of these peaks decreases with increasing the number of scans but no polymeric film was obtained on Pt plate electrode by recording a repetitive CVs scanning. The CVs of **D2** are presented in Figure 4(b) and are characterized by two oxidation peaks at *E*_ox1_ = 0.65 V, *E*_ox2_ = 0.80 V and two reduction peaks at *E*_red1_ = 0.64 V and *E*_red2_ = 0.45 V. The second oxidation peak (*E*_ox2_) is attributed to the strong conjugated electron-donating moiety (N,N-dimethylaniline). During the repetitive scanning of CVs of compound** D2, **the first oxidation (*E*_ox1_) decreases with increasing the number of scans and the potential value for the second oxidation (*E*_ox2_) is shifted in the positive direction. The current intensity decreases by increasing the number of scans. A very thin film of polymer with rainbow color is deposited on Pt plate electrode after repetitive CVs scanning, which is insoluble in methylene chloride and acetone.

In Figure 4(c), the CVs of compound **D3 **in methylene chloride solution, obtained during the successive scans between 0.0 and 1,5 V versus Ag/AgCl, are presented. The CV shows three oxidation peaks at *E*_ox1_ = 0.93 V, *E*_ox2_ = 1.15 V, and *E*_ox3_ = 1.33 V and two reduction peaks at *E*_red1_ = 1.00 V and *E*_red2_ = 0.80 V. The oxidation peaks are associated with the loss of an electron from the atomic central nitrogen of TPA moieties, leading to the formation of cation-radicals. Intensity of these peaks increases with increasing the number of scans. Thus, a green thin film of polymer on Pt plate electrode was deposited by repetitive CVs scans. 

Compound** D4** undergoes quasireversible process exhibits two oxidation peaks at *E*_ox1_ = 0.80 V, *E*_ox2_ = 1.10 V and two reduction peaks at *E*_red1_ = 0.91 V and *E*_red2_ = 0.77 V. The oxidation peaks are assigned to the formation of cation-radicals of triphenylamine at the ends. The first oxidation peak (*E*_ox1_) disappears with increasing the number of scans; the same thing happened with the reduction peak (*E*_red2_). In both cases, the intensity of the current increases with the number of scans; this indicated that a new electroactive structure is formed during the repetitive CV scans. Thus, a green-red colored polymer film is formed and deposited on the Pt plate electrode surface, which is insoluble in methylene chloride and acetone.

**Figure 5:**
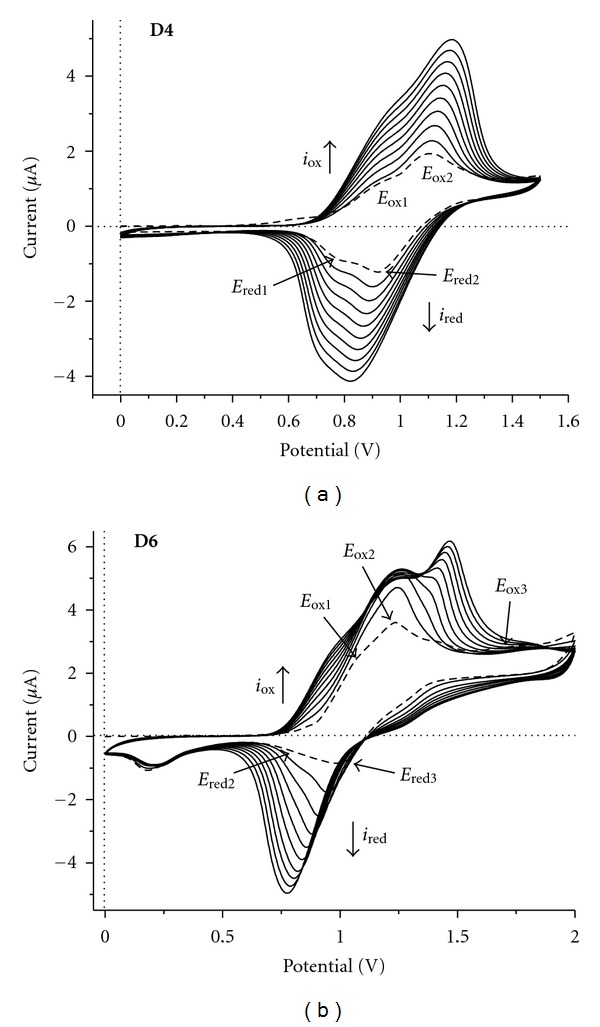
Cyclic voltammograms for compound **D4** (a) and **D6 **(b), 10^−3 ^M, using Bu_4_NBF_4_ as support electrolyte (10^−1 ^M). Scan rate: 50 mV/s between 0.0 and 2.01 V versus Ag/AgCl.

The dendrimer** D6** exhibits three oxidation peaks at *E*_ox1_ = 1.08 V, *E*_ox2_ = 1.23 V, and *E*_ox3_ = 1.43 V and two reduction peaks at *E*_red1_ = 1.023 V and *E*_red2_ = 0.173 V. The three oxidation peaks correspond to the formation of cation-radicals of triphenylamine units (internal and peripheral units) that are formed during the process. Recording the CVs by running a higher number of scans was observed that a third oxidation peak (*E*_ox3_) is shifted in the positive direction. By scanning in the opposite direction the value of reduction peak one (*E*_red1_) is shifted in negative direction with increasing the number of scans. The intensity of the peak current increased regularly during the successive scans (Figure 5(b)). Finally, a green-yellow polymer film deposited on the Pt plate electrode surface was obtained.

The anodic oxidation of triphenylamine and its derivatives was extensively studied starting from 1966 [39–42]. As a conclusion of these studies, TPA-based oligomers fall in the category of multistep processes involving electrochemical-chemical-electrochemical (ECE) reactions. In the first step, a cation radical is formed (TPA^*∙*+^) by oxidation and this is not stable and dimerizes to tetraphenylbenzidine. If all phenyl groups of TPA derivative are tri-para-substituted, the only reaction is the reversible one-electron transfer. In our case, oligomers contain TPA as internal and/or peripheral group. Therefore, only cation-radicals of peripheral TPA are unstable and could dimerize to form tetraphenylbenzidine derivatives, and all processes can be continued until polymer films are deposited on electrode surface. 

According to the correlation which can be done, between HOMO and LUMO energy levels and redox potentials, the value of the energy levels can be calculated from CVs using the on-set values of the oxidation and reduction peaks. The on-set values are estimated from the intersection of the two tangents drawn at the rising oxidation (or reduction) current and the background current in the CVs. The electrochemical data are summarized in Table 2.

Thermal properties of **D1**–**D4** and **D6** were investigated by thermogravimetric analysis (TGA) and differential scanning calorimetry (DSC). TG curves revealed that oligomers were thermally stable materials and the onset decomposition temperatures occurred above 250°C under nitrogen. According to DSC analysis, all compounds remained in amorphous glass state after first heating scan because triphenylamine units have a noncoplanar conformation that hindered the crystallization process.

## 4. Conclusion

In conclusion, we have synthesized six-conjugated arylenevinylene oligomers containing triphenylamine and benzene as core by Wittig and Heck polycondensation methods. The oligomers showed very good solubility in common organic solvents due to the star conformation. All conjugated oligomers exhibit interesting optical properties (absorption and emission) and they also have high fluorescence, which indicate that they are good candidates for the application in OLED as light emitting materials. Cyclic voltammetry was carried out in order to obtain information about the electrochemical stability and the redox process of oligomers. By increasing the *π*-conjugation length, which implies a higher electronic delocalization, the redox behavior of the compounds is changed. It was observed that oligomers containing peripheral TPA groups are able to be electrochemically polymerized by dimerization, when thin polymer films were deposited on working electrode. 

## Figures and Tables

**Table 1:** Optical data for synthesized arylenevinylene oligomers.

Oligomer	*λ*_abs_^max⁡^ (nm)^(a)^	*λ*_em_^max⁡^ (nm)^(a)^	*E*_*g*_(eV)^(b)^
**D** **1**	258, 406	522	2.52
**D2**	325, 404	463	2.75
**D3**	308, 330, 406	465	2.71
**D4**	302, 374	443	2.92
**D5**	316, 374	432	2.94
**D6**	310, 376	445	2.94

^(a)^Determined in diluted CHCl_3_ solution; ^(b)^obtained from UV-Vis spectra: *E*_*g*_ = 1240/*λ*_onset_.

**Table 2:** Electrochemical characteristics of oligomers.

Dendrimer	*E*_ox_^onset^ (V) versus Ag/AgCl	*E*_red_^onset^ (V) versus Ag/AgCl	*E*_HOMO_ (eV)	*E*_LUMO_ (eV)	*E*_*g*_^a^ (eV)
**D** **1**	0.73	1.21	−5.07	−3.13	1.94
**D2**	0.60	0.68	−4.94	−3.66	1.28
**D3**	0.76	1.13	−5.10	−3.21	1.89
**D4**	0.77	1.07	−5.11	−3.27	1.84
**D6**	0.89	1.13	−5.23	−3.21	2.02

^a^ *E*_*g*_ = *E*_HOMO_ − *E*_LUMO_.

## References

[1] Skotheim TA, Reynolds JR (2007). *Handbook of Conducting Polymers*.

[2] MacDiarmid AG (2001). Synthetic metals: a novel role for organic polymers (Nobel lecture). *Angewandte Chemie, International Edition*.

[3] Heeger AJ (2001). Semiconducting and metallic polymers: the fourth generation of polymeric materials (Nobel lecture). *Angewandte Chemie, International Edition*.

[4] Heinze J (1990). Electronically conducting polymers. *Topics in Current Chemistry*.

[5] Higgins SJ (1997). Conjugated polymers incorporating pendant functional groups-synthesis and characterization. *Chemical Society Reviews*.

[6] Noda T, Ogawa H, Noma N, Shirota Y (1999). Organic light-emitting diodes using a novel family of amorphous molecular materials containing an oligothiophene moiety as colour-tunable emitting materials. *Journal of Materials Chemistry*.

[7] Otsubo T, Aso Y, Takimiya K (2002). Functional oligothiophenes as advanced molecular electronic materials. *Journal of Materials Chemistry*.

[8] Nakanishi H, Aso Y, Otsubo T (1999). The longest class of oligothiophenes. *Synthetic Metals*.

[9] Sek D, Grabiec E, Janeczek H (2010). Structure-properties relationship of linear and star-shaped imines with triphenylamine moieties as hole-transporting materials. *Optical Materials*.

[10] Tomalia DA, Naylor AM, Goddard WA (1990). Starbust dendrimers: molecular-level control of size, shape, surface chemistry, topology, and flexibility from atoms to macroscopic matter. *Angewandte Chemie, International Edition*.

[11] Kraft A, Grimsdale AC, Holmes AB (1998). Electroluminescent conjugated polymers—seeing polymers in a new light. *Angewandte Chemie, International Edition*.

[12] D’Andrade BW, Forrest SR (2004). White organic light-emitting devices for solid-state lighting. *Advanced Materials*.

[13] Jenekhe SA (1995). Excited-state complexes of conjugated polymers. *Advanced Materials*.

[14] Miller JS (1993). Conducting polymers—materials of commerce. *Advanced Materials*.

[15] Carroll RL, Gorman CB (2002). The genesis of molecular electronics. *Angewandte Chemie, International Edition*.

[16] Yu G, Gao J, Hummelen JC, Wudl F, Heeger AJ (1995). Polymer photovoltaic cells: enhanced efficiencies via a network of internal donor-acceptor heterojunctions. *Science*.

[17] Cheng YJ, Yang SH, Hsu CS (2009). Synthesis of conjugated polymers for organic solar cell applications. *Chemical Reviews*.

[18] Bhawalkar JD, He GS, Prasad PN (1996). Nonlinear multiphoton processes in organic and polymeric materials. *Reports on Progress in Physics*.

[19] Schon JH, Dodabalapur A, Kloc C, Batlogg B (2000). A light-emitting field-effect transistor. *Science*.

[20] Louie J, Hartwig JF, Fry AF (1997). Discrete high molecular weight triarylamine dendrimers prepared by palladium-catalyzed amination. *Journal of the American Chemical Society*.

[21] O’Regan B, Grätzel M (1991). A low-cost, high-efficiency solar cell based on dye-sensitized colloidal TiO_2_ films. *Nature*.

[22] Zheng M, Bai F, Zhu DJ (1999). New light emitting materials: alternating copolymers with hole transport and emitting chromophores. *Journal of Applied Polymer Science*.

[23] Lai G, Bu XR, Santos J, Mintz EA (1997). Reinvestigation of the Vilsmeier-Haack formylation of triphenylamine. *Synlett*.

[24] Mallegol T, Gmouh S, Meziane MAA, Blanchard-Desce M, Mongin O (2005). Practical and efficient synthesis of tris(4-formylphenyl)amine, a key building block in materials chemistry. *Synthesis*.

[25] Grigoras M, Stafie L (2009). Synthesis and characterization of linear, branched and hyperbranched triphenylamine-based polyazomethines. *Designed Monomers and Polymers*.

[26] Vacareanu L, Grigoras M (2011). Synthesis and electrochemical characterization of new linear conjugated arylamine copolymers. *High Performance Polymers*.

[27] Kuwabara Y, Ogawa H, Inada H, Shirota Y (1994). Thermally stable multilayered organic electroluminescent devices using novel starburst molecules, 4,4′,4^*″*^-tri(N-carbazolyl)triphenylamine (TCTA) and 4,4′,4^*″*^-tris(3-methylphenylphenyl-amino)triphenylamine (m-MTDATA), as hole-transport materials. *Advanced Materials*.

[28] Yeh KM, Lee CC, Chen Y (2008). Poly(4-vinyltriphenylamine): optical, electrochemical properties and its new application as a host material of green phosphorescent Ir(ppy)3 dopant. *Synthetic Metals*.

[29] Saito H, Ukai S, Iwatsuki S, Itoh T, Kubo M (1995). Synthesis of soluble poly(arylenevinylene)s carrying various heterocycles as arylene units. *Macromolecules*.

[30] Zhang X, Yu X, Sun Y (2006). Synthesis and nonlinear optical properties of two new two-photon initiators: triphenylamine derivatives. *Optical Materials*.

[31] Campaigne E, Archer WL (1953). The use of dimethylformamide as a formylation reagent. *Journal of the American Chemical Society*.

[32] Stewart FHC (1960). The Preparation of some surface active alcohols containing the anthracene nucleus. *Australian Journal of Chemistry*.

[33] Sander R, Stuempflen V, Wendorff JH, Greiner A (1996). Synthesis, properties, and guest-host systems of triphenylamine-based oligo(arylenevinylene)s: advanced materials for LED applications. *Macromolecules*.

[34] Zhang X, Yu X, Yao J, Jiang M (2008). Synthesis and nonlinear optical properties of two three-branched two-photon polymerization initiators. *Synthetic Metals*.

[35] Plater MJ, Jackson T (2003). Polyaromatic amines. Part 3: synthesis of poly(diarylamino)styrenes and related compounds. *Tetrahedron*.

[36] Heller A (1964). Organic liquid scintillators. VI. Substituted distyrylbenzenes: scintillation properties and spectra of absorption and fluorescence. *The Journal of Chemical Physics*.

[37] Wang HY, Chen G, Xu XP, Chen H, Ji SJ (2011). The synthesis and photophysical properties of novel poly(diarylamino) styrenes. *Dyes and Pigments*.

[38] Jiang Y, Wang JY, Ma Y, Cui YX, Zhou QF, Pei J (2006). Large rigid blue-emitting *π*-conjugated stilbenoid-based dendrimers: synthesis and properties. *Organic Letters*.

[39] Seo ET, Nelson RF, Fritsch JM, Marcoux LS, Leedy DW, Adams RN (1966). Anodic oxidation pathways of aromatic amines. Electrochemical and electron paramagnetic resonance studies. *Journal of the American Chemical Society*.

[40] Creason SC, Wheeler J, Nelson RF (1972). Electrochemical and spectroscopic studies of cation radicals. I. Coupling rates of 4-substituted triphenylaminium ions. *Journal of Organic Chemistry*.

[41] Chiu KY, Su TX, Li JH, Lin TH, Liou GS, Cheng SH (2005). Novel trends of electrochemical oxidation of amino-substituted triphenylamine derivatives. *Journal of Electroanalytical Chemistry*.

[42] Vacareanu L, Grigoras M (2010). Electrochemical characterization of arylene vinylene oligomers containing triphenylamine and carbazole units. *Journal of Applied Electrochemistry*.

